# Fingolimod modulates microglial activation to augment markers of remyelination

**DOI:** 10.1186/1742-2094-8-76

**Published:** 2011-07-05

**Authors:** Samuel J Jackson, Gavin Giovannoni, David Baker

**Affiliations:** 1 Centre for Neuroscience and Trauma, Blizard Institute of Cell and Molecular Science, Barts and the London School of Medicine and Dentistry, Queen Mary University of London, London, United Kingdom

## Abstract

**Introduction:**

Microglial activation in multiple sclerosis has been postulated to contribute to long-term neurodegeneration during disease. Fingolimod has been shown to impact on the relapsing remitting phase of disease by modulating autoreactive T-cell egress from lymph organs. In addition, it is brain penetrant and has been shown to exert multiple effects on nervous system cells.

**Methods:**

In this study, the impact of fingolimod and other sphingosine-1-phosphate receptor active molecules following lysophosphotidyl choline-induced demyelination was examined in the rat telencephalon reaggregate, spheroid cell culture system. The lack of immune system components allowed elucidation of the direct effects of fingolimod on CNS cell types in an organotypic situation.

**Results:**

Following demyelination, fingolimod significantly augmented expression of myelin basic protein in the remyelination phase. This increase was not associated with changes in neurofilament levels, indicating de novo myelin protein expression not associated with axonal branching. Myelin wrapping was confirmed morphologically using confocal and electron microscopy. Increased remyelination was associated with down-regulation of microglial ferritin, tumor necrosis factor alpha and interleukin 1 during demyelination when fingolimod was present. In addition, nitric oxide metabolites and apoptotic effectors caspase 3 and caspase 7 were reduced during demyelination in the presence of fingolimod. The sphingosine-1-phosphate receptor 1 and 5 agonist BAF312 also increased myelin basic protein levels, whereas the sphingosine-1-phosphate receptor 1 agonist AUY954 failed to replicate this effect on remyelination.

**Conclusions:**

The results presented indicate that modulation of S1P receptors can ameliorate pathological effectors associated with microglial activation leading to a subsequent increase in protein and morphological markers of remyelination. In addition, sphingosine-1-phosphate receptor 5 is implicated in promoting remyelination in vitro. This knowledge may be of benefit for treatment of chronic microglial inflammation in multiple sclerosis.

## Introduction

Multiple sclerosis (MS), the prototypic inflammatory demyelinating disease of the central nervous system (CNS), is considered an autoimmune disorder with a secondary neurodegenerative component with associated oligodendrocyte pathology. During the relapsing-remitting disease course evident in the majority of patients, inflammation is the key driver of disease, with inflammatory infiltration correlating with bouts of clinical symptoms. The typical course changes later in disease, becoming progressive in nature and lacking periods of remission. This secondary progressive phase lacks many of the markers of immune involvement seen in earlier disease, but displays ongoing demyelination and axonal loss, which results in a progressive functional decline [1]. Therapies currently available to people affected by MS target the immune-related component of the disease, and none have yet been shown to convincingly impact on the secondary neurodegenerative phase [2]. It has been postulated that one mechanism for neuroprotection in MS would be remyelination, especially given that this is the natural reparatory mechanism following a relapse in MS, and in an animal model, experimental autoimmune encephalomyelitis (EAE; [3]). As well as restoring saltatory conduction, remyelination forms a physical barrier to secondary axonal degeneration in the lesion environment. Direct damage to neurons and neuronal apoptosis can be caused by excitotoxic mediators, neurotoxic cytokines and free radical species present in chronic MS lesions, in spite of the reduced inflammatory infiltrate [4].

A compound recently licensed for treatment of relapsing forms of MS, fingolimod (FTY720, approved as Gilenya™ in US and several other countries), is a sphingosine-1-phosphate (S1P) receptor modulator that can cause retention of T-cells in lymph organs when phosphorlyated to the active form [5]. It is a potent agonist at S1P1, 4 and 5 receptors, less so at S1P3, and has minimal activity at S1P2 [5,6]. In addition, fingolimod can act as a functional antagonist at S1P1 receptors in lymphocytes by inducing their internalisation and subsequent degradation [7]. By this mechanism, the compound reduces immune cell infiltration into the CNS, with Phase III trials demonstrating effects on MRI activity, brain atrophy and relapse rate [8]. It has been proposed that fingolimod may also directly affect cells of the CNS [9]. It is blood-brain barrier penetrant due to its lipophilic nature, can reach physiologically meaningful concentrations in CNS tissue and preferentially localizes to myelinated tracts [10]. S1P receptors are expressed on all CNS cell types, providing a basis for direct CNS effects. S1P receptor subtype specific agonists have also been synthesized by Novartis: AUY954 is active at S1P1 receptors [11] and is a tool compound, and BAF312 is active at S1P1 and S1P5 receptors [12,13] and has completed phase II clinical trials in MS [8].

*In vitro *studies on single cell types have identified a range of effects on CNS cells. S1P signaling in oligodendrocytes has been shown to play roles in cell survival [14], proliferation [15] and process dynamics [16]. In culture, activation of S1P receptor with fingolimod protected oligodendrocytes against apoptosis mediated by growth factor deprivation in a mechanism linked to extracellular signal-regulated kinase (ERK) 1/2 and akt phosphorylation [14]. ERK phosphorylation has been shown to be pro-survival in a number of relevant paradigms, including in apoptosis mediated by glial-derived reactive oxygen species. The relatively high concentration of fingolimod used in this study also elicited arrest of oligodendrocyte differentiation, which was reversed by neurotrophin 3 [17]; fingolimod in lower doses does not arrest differentiation [15]. Fingolimod can also play a role in platelet derived growth factor-induced oligodendrocyte precursor cell (OPC) mitogenesis, in an effect elicited by S1P1.

Oligodendrocyte process modulation by fingolimod has been demonstrated in OPCs [16] and mature human oligodendrocytes [18] in a time-dependent process. Short-term fingolimod treatment of OPCs in culture caused process retraction, while longer term treatment led to process extension and enhanced survival [16]. These studies demonstrated that S1P1 and S1P5 mRNA transcripts are modulated by fingolimod in a cyclical and reciprocal manner, leading to the time-dependent effect. Finally, fingolimod has been shown to increase remyelination when assessed morphologically in brain slice cultures [19].

A microglial response to fingolimod has been postulated, but this is as yet unclear. In a model of traumatic brain injury [20] and of ischemia [21], fingolimod reduced microglial activation as assessed by immunohistochemistry. In the traumatic brain injury model, this was associated with a reduction in apoptosis linked to reduced microglia-derived interleukin 16 [22]. However, others have found that non-cytotoxic microglial numbers are increased by fingolimod, but that their activation status remained unchanged [19].

Fingolimod has been shown to be cytotoxic to neurons, at high concentrations *in vitro *[23]. At lower concentrations this toxicity was absent, and no evidence has been produced that neuronal degeneration occurs in other paradigms using the compound. In astrocytes, ERK phosphorylation following fingolimod treatment was shown [24]. Previously, ERK phosphorylation in astrocytes has been shown to cause proliferation, though this was not demonstrated in the study cited. Astrocyte migration can also be elicited through S1P receptor signaling [25].

The compound has also been shown to reduce clinical signs in EAE, with the effects linked to reduced systemic and CNS inflammation. Two separate studies in the DA rat examined this from different viewpoints: gene expression [26] and cellular inflammatory markers [27]. Genes encoding inflammatory mediators and vascular adhesion molecules were down-regulated by treatment with fingolimod. In addition, expression of matrix metalloproteinase 9 and tissue inhibitor of metalloproteinase 1 were reduced, indicating a role for fingolimod in maintenance of blood-brain barrier integrity. The second study confirmed an attenuation of inflammatory mediators including interferon gamma and nitric oxide (NO), due to a reduction in CNS penetrant lymphocytes. Similar effects have been demonstrated in other EAE models, in Lewis rat [28], SJL mice [29] and C57BL/6 mice [30].

The reaggregate spheroid cell culture model employed in this study myelinates over 25 days in vitro, and can be demyelinated using lysophosphatidyl choline, following which spontaneous remyelination occurs [31,32]. Pro-remyelinative effects can be elucidated by observing augmentation of this remyelination. The model is devoid of classical blood-borne immune cells, but contains CNS-resident microglia (5-10% of total cell population [33]); therefore, effects on the CNS can be elucidated in the absence of the immune system.

In this study we provide evidence for direct effects of fingolimod on remyelination via dampening of the microglial response, associated with a reduction in apoptosis and modulation of cytokine levels in the reaggregate spheroid cell culture model.

## Materials and methods

### Animals

All animal experiments were performed in accordance with the UK Animals (Scientific Procedures) Act 1984. Time mated pregnant Sprague Dawley rats were obtained from Charles River, Margate, UK.

### Media and compounds

Mechanical dissociation and subsequent washes % centrifugation were carried out in D1 solution [34], containing 138 mM NaCl, 5.4 mM KCl, 0.17 mM Na_2_HPO_4_, 0.22 mM KH_2_PO_4_, 5.55 mM D-glucose, 58.43 mM sucrose, 5 mg%L phenol red (Invitrogen, Paisley, UK). Aggregates were cultured in high glucose DMEM supplemented with 10% fetal bovine serum (Invitrogen) and 100 U/ml penicillin-streptomycin (Invitrogen) as described previously [31]. After 5 days in culture, medium was further supplemented with 0.5 mM n-acetyl cysteine (Sigma Aldrich, Poole, UK). Fingolimod, AUY954 and BAF312 were supplied by Novartis Pharmaceuticals, Basel, CH.

### The rat CNS aggregate cell culture system

Fetuses were removed at embryonic age 16 days following sacrifice of the adult by decapitation, and placed in sterile, ice-cold D1 solution. The telencephalon was dissected from each and pooled in ice-cold sterile D1 solution. After washing twice in D1 solution, brain tissue was progressively dissociated by sieving through a 200- μm followed by a 115- μm pore nylon mesh (Plastok, Liverpool, UK). The filtrates were centrifuged at 300 *g *for 15 min at 4°C, tested for viability using 0.1% trypan blue exclusion, and re-centrifuged. The remaining telencephalon cell population was seeded at 4 × 10^7 ^cells per flask and incubated at 37°C in a humidified 9% CO_2_/91% O_2 _atmosphere (Heraeus incubator, Philip Harris Scientific, Ashby-de-la-Zouch, UK) under constant rotation at 83 rpm (Kuhner Shaker, Philip Harris Scientific). The day of seeding was termed day *in vitro *zero (DIV 0). Cells were transferred to larger flasks on DIV 2, and the DMEM volume doubled to 8 ml. On DIV 5, 8, 11, 14 and subsequently every other day, cultures were fed by replacing 5 ml of pre-warmed DMEM in each flask. Myelin basic protein is seen following DIV 14, and increases throughout the culture period, as does neuronal marker neurofilament. This is in line with development in the fetal brain at this time-point post-partum [35,36], and reflects the developmental nature of this model.

### Demyelination and treatment protocol

0.14 mM lysophosphotidyl choline (Sigma) was added to pre-warmed culture medium. Cultures were maintained as described, substituting medium for lysophosphotidyl choline-containing medium, on DIV 25 and 27. Lysophosphotidyl choline was removed by medium replacement three times on DIV 29. Control flasks were also subjected to triple medium replacement. Treatment with fingolimod, AUY954 or BAF312 commenced on DIV23 and continued until DIV40. 3 μM fingolimod, AUY954 or BAF312 was added to the culture medium on each media replacement. Fingolimod was also tested at a concentration of 1 and 10 nM, as effects had been seen previously at low doses [19]. However, these concentrations elicited no changes in myelin basic protein or microglial activation (data not shown). The concentration used was therefore based on that of fingolimod observed in the brain of lewis rats with EAE which were given the compound [10]. Measured concentrations were around 500 ng/g of tissue, which is equivalent to around 1.5 μM. This was doubled to account for non-specific binding to protein in the serum-containing medium.

### Sampling of aggregates

For confocal microscopy, aggregates were fixed in 4% paraformaldehyde in phosphate buffer, pH 7, for 2 hours prior to washing and storage at 4°C in PBS containing 0.01% sodium azide. For analysis by ELISA or western blot, aggregate samples were homogenized by sonication for 30 s in Tissue Protein Extraction Reagent (T-PER; Pierce) with protease inhibitors (20 μg/ml leupeptin, 20 μg/ml pepstatin A, 200 μg/mlbenzoylarginine methyl ester, 200 μg/mltosylarginine methyl ester, 200 μg/ml trypsin inhibitor, 200 μg/mltosylphenylalanyl chloromethane, 200 μg/ml aprotinin and 4 mM ethyl glycol tetra-acetic acid). Total protein was assayed using the BCA kit (Thermo Fisher, Cramlington, UK) with bovine serum albumin as a standard. For analysis of cytokines on the Mesoscale Discovery platform, culture medium was sampled, frozen on dry ice and stored at -20°C.

### Confocal microscopy

Free-floating whole aggregates were stained by incubation overnight at 4°C under constant inverting rotation with a relevant primary antibody (goat anti-NFH, AbCam, Cambridge, UK) in phosphate buffered saline plus 0.1% Tween-20 (PBS-T). After washing three times for 1 hour under constant inverting rotation with PBS-T, aggregates were labeled with a second primary antibody overnight (rabbit anti-MBP, AbCam) using the same method. Following a further wash, relevant fluorochrome-conjugated secondary antibodies were applied and aggregates incubated overnight as previously. After immunolabelling, aggregates were placed on a glass microscope slide (VWR International Ltd, Lutterworth, UK) followed by a drop of PBS/glycerol (1:8). A glass cover slip was then placed on top of the aggregates, and sealed. Aggregates were analysed on a Zeiss LSM laser scanning confocal microscope.

### Measurement of myelin basic protein, neurofilament H, ferritin and nitric oxide by ELISA

Nitric oxide was measured using a Total Nitric Oxide and Nitrate/Nitrite Parameter Assay Kit according to manufacturer's instructions (R&D Systems, Abingdon, UK) following filtration of samples to remove high molecular weight protein components using a centrifugal device (Millipore, Watford, UK). Antibodies used for the myelin basic protein (MBP, [37]), neurofilament (Nf, [37]) and ferritin (Fe; [38]) ELISAs have been described previously. Coat antibody was diluted in coating buffer (100 mm Na_2_HPO_4 _adjusted to pH 9.0 with 100 mm NaH_2_PO_4_), and 100 μL was added to each well of a 96-well NuncMaxisorp plate (VWR International). The plate was then incubated overnight at 4°C. After bringing the plate to room temperature, it was washed once using PBS-T (Sigma). Non-specific binding was blocked by incubation with 1% bovine serum albumin (Sigma) in PBS-T for 1 hour at room temperature. Following blocking, the plate was washed once using PBS-T and then incubated with diluted samples and standards for 2 h at room temperature under gentle agitation. After washing four times as above, the detect antibody was applied at 1:1000 and the plate incubated as previously for 1 h. For MBP and Nf, horseradish peroxidase-conjugated reporter antibody was applied at 1:1000 and the plate was incubated for 1 h at room temperature. The secondary antibody in the ferritin ELISA is directly conjugated to HRP, so this was not necessary for this analyte. TMB Substrate (R and D Systems UK Ltd, Abingdon, UK) was applied following four final washes, and the colour developed for 15 min before being stopped with 1 m phosphoric acid. The plate was read on a photometer (AnthosLabtec Instruments, Salzburg, Austria) at 450 nm wavelength, using 620 nm wavelength as a reference measurement.

### Mesoscale Discovery Platform multiplex cytokine measurements

To assess the concentrations of cytokines in the culture medium samples were assessed using the Mesoscale Discovery Platform multiplex system, according to manufacturer's instructions. Briefly, non-specific binding on the plate was blocked with bovine serum albumin and sample added to each well. Following incubation, washing, and secondary antibody cocktail incubation, the plate was read on a MSD SECTOR Imager 2400.

### Measurement of Caspase 3 Western blotting

Proteins were separated using sodium docylsulphate polyacrylamide gel electrophoresis using a mini-protean system (Bio-Rad, Hemel Hempstead, UK). Protein was run on a 12% tris-glycine gel (BioRad), transferred to nitrocellulose membrane, blocked with 5% fat-free milk and probed with the antibodies active and pro caspase 3 (AbCam). Following incubation with an appropriate HRP-conjugated secondary antibody (Dako, Ely, UK), visualisation was carried out using the ECL detection system (GE Healthcare, Little Chalfont, UK), and the membrane was exposed to radiological film. Band density was measured using computer, and normalised to GAPDH as a loading control.

### Electron Microscopy for identification of myelin structures

Spheroids were washed with sterile PBS (Gibco) and fixed for 3 hours in EM fixative (2% Paraformaldehyde 2.5% gluteraldehyde in 0.1 M phosphate buffer) before being post fixed for 2 hours in osmium tetroxide. Following embedding in araldite resin (Agar), 1 micron sections were cut and stained, and viewed on a Hitachi H7600 transmission electron microscope.

## Results

### Fingolimod increases myelin basic protein following a demyelinative insult

**Figure 1:**
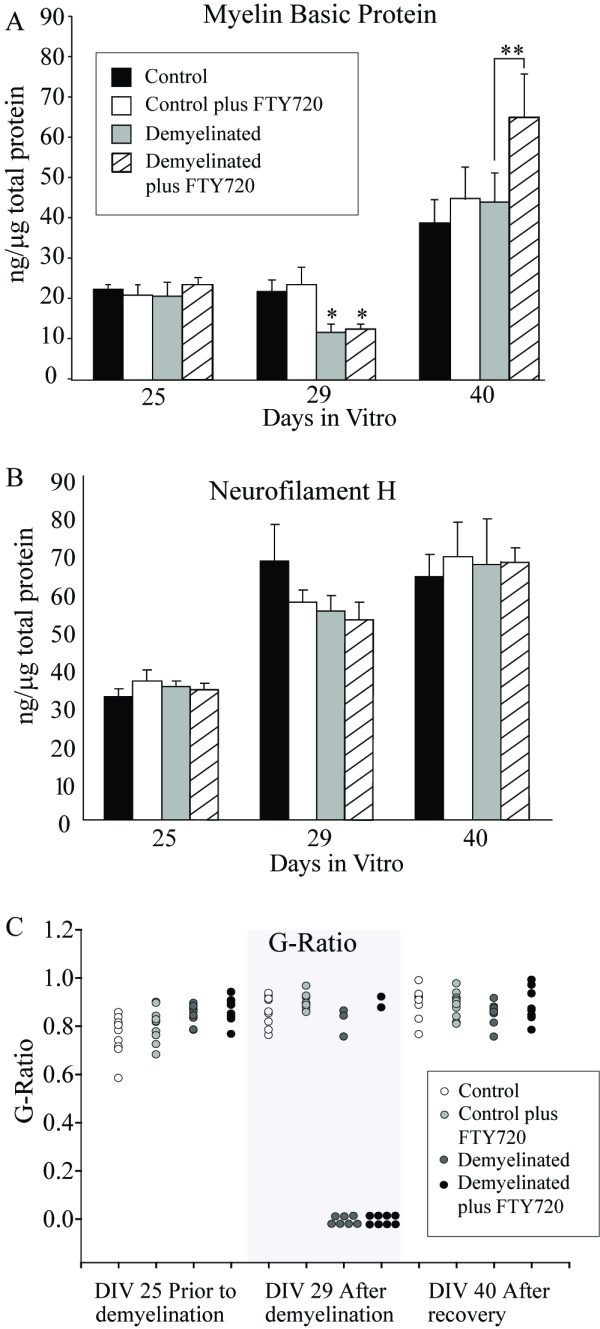
**Spheroid myelinating cultures were prepared from fetal rat telencephalon and demyelinated with lysophosphotidyl choline on DIV 25-29 in the presence or absence of fingolimod**. Myelin basic protein (A) and neurofilament (B) levels were measured using protein specific ELISA following demyelination. C) Spheroids were fixed and processed for electron microscopy. The mean ratio (+/- SEM) of fibre diameter to myelin thickness was determined (G-ratio) in a minimum of 10 fibres per sample from 3 samples per group. Results were analysed by ANOVA with Holm-Sidak post test. * = p < 0.05; ** = p < 0.01.

**Figure 2:**
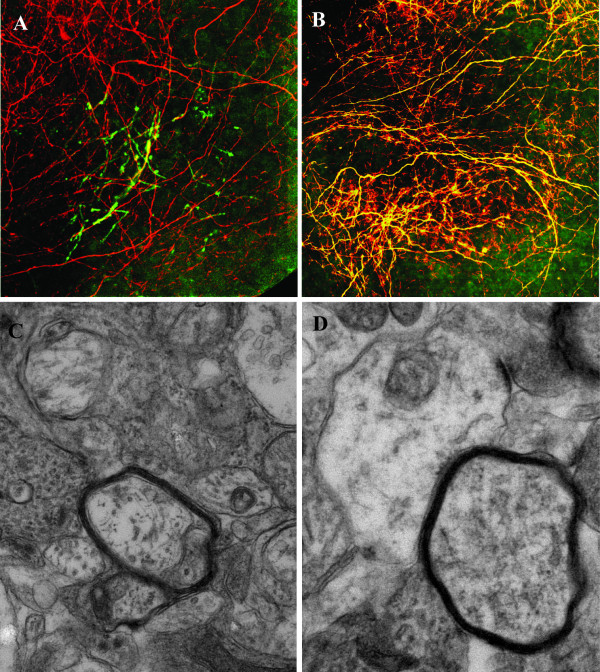
**Spheroid myelinating cultures were prepared from fetal rat telencephalon and demyelinated with lysophosphotidyl choline on DIV 25-29 in the presence or absence of fingolimod**. Confocal and electron microscopy was carried out to determine morphology of myelinated fibres. Spheroids were fixed and stained whole with specific antibodies against myelin basic protein (green) and neurofilament (red) after demyelination (A) and following remyelination (B). Spheroids prepared for electron microscopy were imaged to assess morphology prior to demyelination (C) and following remyelination (D).

Myelinated neurospheres were demyelinated by transient treatment with lysophosphotidyl choline from DIV 25 to DIV 29. Neurofilament levels as measured by ELISA did not significantly change over the demyelination period or following recovery, indicating that axonal damage was not a significant component of pathology in this model (Figure 1). This also indicates that the observed MBP level increase was not due to myelination of fingolimod-induced *de novo *neuronal sprouting. The trend towards increasing NF levels can be attributed to the developmental nature of the model. Myelin basic protein expression was measured by ELISA, and fluorescent staining for MBP in spheroids was analysed using laser scanning confocal microscopy, as a surrogate marker for myelination status. MBP levels were significantly reduced following lysophosphotidyl choline-induced demyelinative insult, and this was not altered by the presence of fingolimod (Figure 1). At DIV 40, MBP levels in demyelinated cultures had returned to that of control, indicating that the spontaneous remyelination associated with this model had occurred. The MBP level in fingolimod treated cultures rebounded beyond the control level, and was significantly higher at this last time point, indicating an enhancement of remyelination. These results were morphologically corroborated by confocal microscopy for MBP and neurofilament (Figure 2).

To confirm the presence of morphologically complete multi-lamellar myelin, electron microscopy was performed on spheroids from each time point. Spheroids were examined for presence of morphological indicators of myelination, demyelination and remyelination. Prior to demyelination, compact myelin could be identified in neurospheres by identification of concentric tightly packed rings of electron dense material, complete with characteristic major dense lines. These were not present following lysophosphatidyl choline treatment, indicating demyelination had occurred similarly in fingolimod treated and control cultures. Following the recovery period, multi-lamellar myelin was once again present in the spheres, indicating remyelination had occurred (Figure 2). The g-ratio of fibre diameter to fibre plus myelin diameter was measured to assess the extent of remyelination, and to identify thin myelin sheaths characteristic of remyelination (Figure 1). G-ratio measurements from pre-demyelinated neurospheres indicated that the g-ratio values agreed with previously published measurements of myelin thickness *in vivo*. However, the range of g-ratio values was extended compared to published *in vivo *values, indicating that in this culture system, myelin thickness and axonal diameter are less closely co-regulated than in the *in vivo *situation. Following remyelination, g-ratio values were not increased, as could be expected from *in vivo *studies, indicating a lack of characteristic remyelinated fibres. There was no significant difference in fingolimod treated cultures compared with control.

### Fingolimod negatively regulates markers of inflammation in the absence of lymphocytes and immune organs

**Figure 3:**
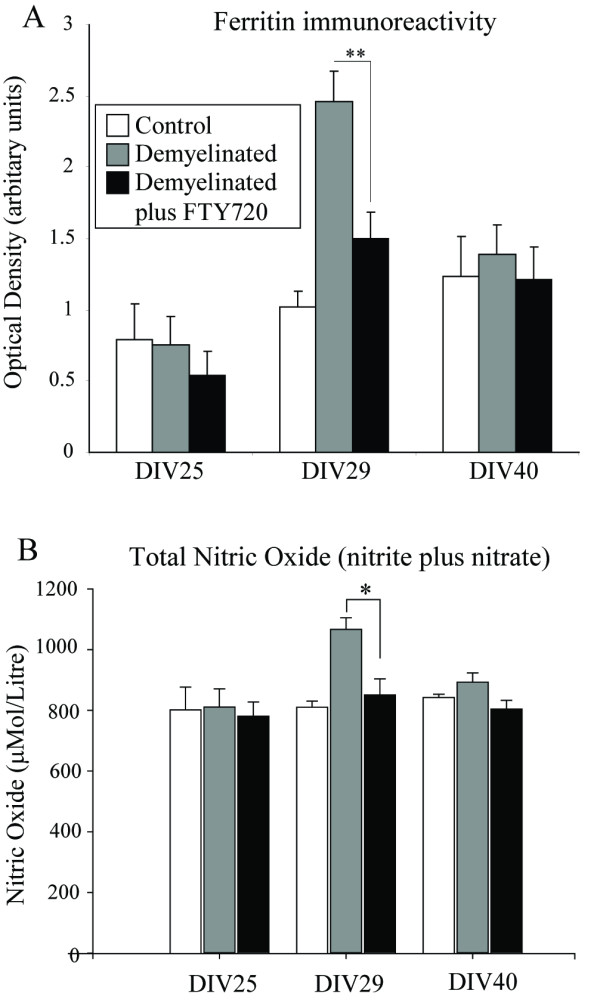
**Spheroid myelinating cultures were prepared from fetal rat telencephalon and demyelinated with lysophosphotidyl choline on DIV 25-29 in the presence or absence of fingolimod**. Samples of spheroids were homogenised in protein extraction buffer, centrifuged and the supernatant removed. Samples were analysed by ELISA for (A) ferritin and (B) nitric oxide over the course of demyelination and remyelination. Results were analysed by ANOVA with Holm-Sidak post test, and are expressed as mean +/- SEM, n = 10. * = p < 0.05; ** = p < 0.01.

In order to evaluate the impact of fingolimod on microglia, an ELISA for ferritin was performed, as increased ferritin levels are indicative of microglial activation (Figure 3A; [38]). Ferritin levels increased following demyelination, and had returned to control levels following remyelination. Fingolimod reduced, but did not abolish, this increase in ferritin immunoreactivity, indicating that the compound partially inhibited the development of microglial activation.

In order to assess the presence of microglia/astrocyte-derived pathological indicators, NO metabolites were measured (Figure 3B). The cultures were found to have a high 'background' level of NO metabolites, which was significantly increased following demyelination. This increase was abolished in fingolimod-treated cultures.

**Figure 4:**
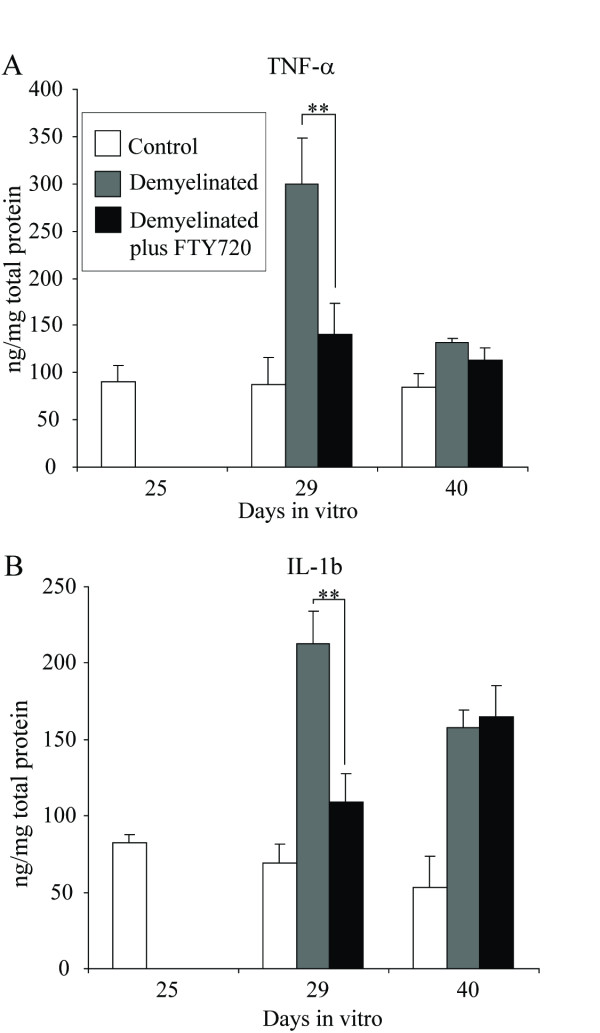
**Spheroid myelinating cultures were prepared from fetal rat telencephalon and demyelinated with lysophosphotidyl choline on DIV 25-29 in the presence or absence of fingolimod**. Multiplex ELISA was used to analyse cytokine production in tissue culture supernatant. Culture medium was snap frozen and subsequently analysed using the Mesoscale Discovery Platform. Mean levels of tumor necrosis factor alpha and interleukin 1b were determined and expressed per milligram total protein +/- SEM (n = 10 per goup). ** = p < 0.01.

In order to examine the impact of fingolimod on the immune microenvironment of the CNS, cytokines involved in the inflammatory process were measured (Figure 4). Demyelination resulted in an increase in the levels of tumor necrosis factor alpha (TNF-α) and interleukin (IL)-1b which was abolished by fingolimod treatment. Following remyelination, IL-1b in lysophosphotidyl choline-treated spheroids were significantly higher than controls, indicating a transient effect of fingolimod. Levels of TNF-α were significantly reduced in all cultures following remyelination.

### Fingolimod reduces apoptosis following a demyelinative insult

**Figure 5:**
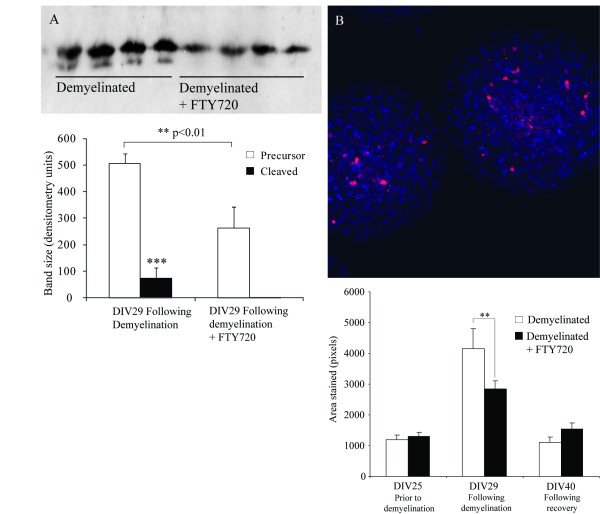
**Spheroid myelinating cultures were prepared from fetal rat telencephalon and demyelinated with lysophosphotidyl choline on DIV 25-29 in the presence or absence of fingolimod**. Pro-caspase 3 and caspase 3 levels were assessed in homogenized spheroids with western blot (A, B) following homogenization and separation on polyacrylamide gel (n = 4; ** = p < 0.01; *** = p < 0.001). Caspase 3 and 7 activity were assessed in intact spheroids using the CaspaTag staining system (C, D) followed by fluorophore conjugation and confocal microscopy (n = 8; ** = p < 0.01). Results were analysed by ANOVA with Holm-Sidak post test.

Demyelinative treatment of spheroids has been shown to induce apoptosis [39]. In order to assess the impact of fingolimod on apoptosis in this system, western blotting was performed for intact (non-activated) and cleaved (activated) caspase 3 following demyelinative insult (Figure 5A). Fingolimod treated cultures expressed less intact caspase 3 than control cultures, none of which was cleaved to the active form. This indicated a significant reduction in caspase 3 production and activation, as was confirmed by using the CaspaTag staining system for caspase 3 and 7 (Figure 5B). Staining with CaspaTag increased following demyelination, and this increase was significantly attenuated by fingolimod. CaspaTag staining fell to previous low levels following the recovery period, suggesting that demyelination-induced apoptosis is a transient event.

### S1P5, but not S1P1, agonism elicits enhanced remyelination in vitro

**Figure 6:**
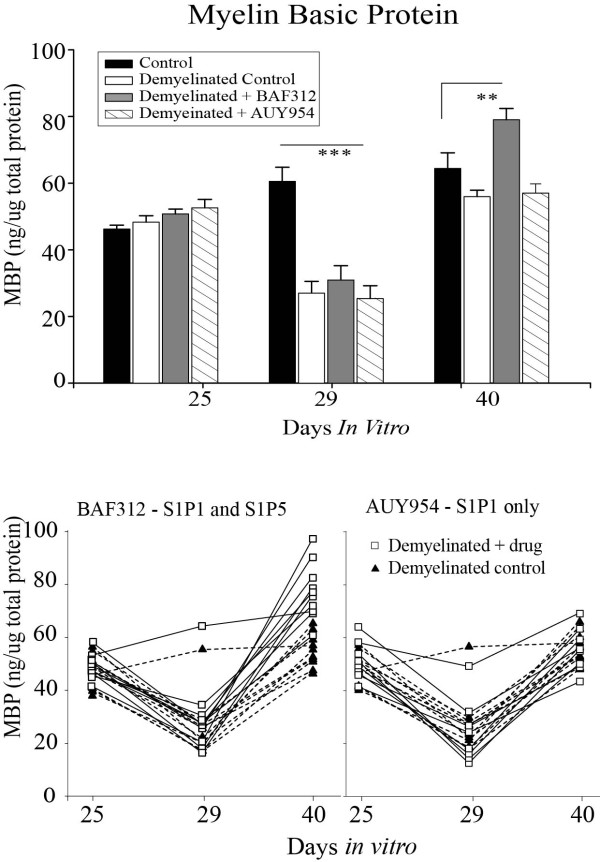
**Spheroid myelinating cultures were prepared from fetal rat telencephalon and demyelinated with lysophosphotidyl choline on DIV 25-29 in the presence or absence of AUY954, an S1P1 agonist, or BAF312, an S1P1 and S1P5 agonist**. Myelin basic protein (A) and neurofilament (B) levels were measured using protein specific ELISA following demyelination. Results were analysed by ANOVA with Holm-Sidak post test. * = p < 0.05; ** = p < 0.01.

Aggregates were seeded, allowed to mature and demyelinated as previously, and treated over the same period with AUY954, active at S1P1, or BAF312, active at S1P1 and S1P5. Aggregates were analysed for MBP and NF content by ELISA. Cultures treated with BAF312 exhibited a significant rebound in MBP levels following demyelination compared with control, whereas cultures treated with AUY954 did not (Figure 6). This indicates that agonism at S1P1 is not sufficient to elicit changes in remyelination, but that this may be mediated through signaling at S1P5. Neurofilament levels were unchanged between groups, and were not affected by the demyelinative insult, indicating that the effect on MBP was not due to de novo axonal sprouting.

## Discussion

This paper describes direct effects of fingolimod on cells of the rat CNS. Here, we demonstrate that fingolimod can actively promote remyelination following an insult elicited by lysophosphotidyl choline, indicating direct interaction of fingolimod with oligodendrocytes, microglia and/or astrocytes.

Fingolimod increased markers of myelination as assessed biochemically and morphologically in a culture model lacking monocytes and other components of the immune system, but including microglia. Fingolimod has been shown to reduce pathology associated with demyelination *in vivo *models, although this can largely be attributed to sequestration of reactive T lymphocytes in lymphoid organs. Fingolimod has also been shown to alter myelin gene expression, and preferentially localises to myelin tracts *in vivo *[10]. In culture, fingolimod exerts dose-dependent effects on OPCs, and can prevent OPC apoptosis and differentiation [16]. OPCs are thought to be intimately involved in the remyelination process in humans, and OPC dysfunction may be one factor preventing remyelination in MS [40]. Oligodendrocyte progenitors and precursors are present in the spheroid cultures, and persist after demyelination [41]. The full range of oligodendrocyte progenitor and precursor markers have been identified by fluorescent immunostaining, and include platelet derived growth factor alpha receptor, NG2 chondroitin sulphate, O4 and galactocerebroside. Early markers decline by the point at which demyelination is induced, but are still present; pre-myelinating O4 and galactocerebroside positive oligodendrocytes are present in higher numbers around the point at which demyelination is induced, providing a source of potentially remyelinating oligodendrocytes.

Interestingly, agonism of S1P receptor 5 increased myelin basic protein levels above those of control, indicating a role for this receptor in remyelination. This effect was not observed using an agonist to S1P1, indicating a heterogenous role for these receptors in remyelination. This agrees with previously published in vitro results, showing that S1P5 activation can induce changes in oligodendrocyte morphology and myelination [15,19]. Differences in G-protein coupled receptor signaling may underlie the hetrogenous effects observed-S1P1 signals using G αi/o only, employing cAMP as a secondary messenger, whereas S1P5 can also signal via G α12/13 which signals via the Rho GTPase [42]. Rho signaling has been shown to regulate oligdendrocyte precursor cell cycle events via a network of positive and negative regulators [16,43].

Lysophosphotidyl choline has been postulated to cause demyelination via several routes. It has been shown to act as a lipodestructive detergent with the ability to preferentially destroy lipid-rich membranes of myelin sheaths. These structures are highly susceptible to ionic detergent activity. Importantly for this study, lysophosphotidyl choline has also been shown to elicit activation of microglia [44] as assessed morphologically [45] and by cytokine production [46], providing another route for demyelination. In addition, specific G-protein receptor and microtubule associated protein kinase-mediated interactions between lysophosphotidyl choline and the oligodendrocyte have been uncovered, opening the potential for ligand mediated cell death. Apoptosis is reduced in this culture system by fingolimod but it is unlikely that fingolimod reduced apoptosis via ligand-mediated effects [47]. It is more likely that reduction of microglia/astrocyte-derived factors and modulation of the pathological milieu was responsible for a reduction in apoptosis. The identity of these apoptotic cells in the spheroids was not probed in this study; this could be examined in primary monolayer cultures where enumeration of apoptotic vs. normal cells could be more effectively performed. Given the paradigm used, the authors predict that oligodendrocytes will be the cells susceptible to apoptosis.

Fingolimod altered the *in vitro *microenvironment via direct effects on microglia and possibly astrocytes. Demyelination resulted in the expression of the cytokines TNF-α and IL1b which were down-regulated by treatment with fingolimod. These cytokines are produced by activated microglia and astrocytes, and are pathological effectors in EAE and MS. Microglial activation in MS is seen acutely during demyelination, and motile activated macrophages can contribute to demyelination or repair. Microglia are also chronically activated in normal appearing white matter during MS, and may contribute to axonal damage. Molecules which modulate microglial activation have been shown to be effective in experimental models of MS, including EAE. In this model, demyelination also caused an up regulation in NO species, albeit against a high background of NO. This significant rise was attenuated by fingolimod, likely by effects on microglia and astrocytes. Given that disease modification with fingolimod is possible in ischemic and brain injury models via dampening microglial response [22,48], it is feasible that fingolimod would also modulate secondary disease in MS models. However, recent data from this group [49] contradicts this hypothesis, with fingolimod having no effect on progressive secondary experimental autoimmune encephalomyelitis. In the acute non-gliotic disease state, fingolimod provides protection in this model, providing evidence to suggest FTY720 will not elicit repair beyond a certain disease stage. In addition, recent data has been published showing that FTY720 does not elicit remyelination in the non-inflammatory cuprizone model, further strengthening the notion that the neuroprotective effects of FTY720 are mediated by dampening the inflammatory process [50].

The findings presented are in agreement with previous published studies. S1P1 receptor activation modulates phosphorylation of ERK, which has been shown to protect oligodendrocytes from microglia-derived, reactive species-mediated apoptosis [14]. In this study, fingolimod was found to down-regulate NO species production following demyelination. In addition, fingolimod has been shown to protect OPCs from microglial conditioned medium induced cell death, mediated by the pro-inflammatory cytokines interferon gamma and TNF-α [51]. Again, fingolimod was shown to modulate IL1b and TNF-α production following insult in this model, indicating effects on microglia. In models of traumatic brain injury, fingolimod has been shown to attenuate major histocompatibility complex II and Endothelial monocyte-activating polypeptide II expression indicating an anti-inflammatory role [22]. In addition, activation of S1P receptors in a rodent model of cerebral ischemia has been shown to protect against neuronal cell death, reducing infarct volume by down-regulation of microglial activation [48]. Microglia and, to a lesser extent, astrocytes have also been shown to produce IL16 in response to immunological challenge, and fingolimod has been shown to be able to reduce production of this cytokine [20].

Myelin basic protein was used as a surrogate marker for myelination in this study, as it is expressed as a late marker of myelination, and is involved in the final stages of myelination - compaction. The protein has been used as a marker of mature myelin in *ex vivo *and *in vitro *material [52], and correlates with myelination in this system [39]. As is the case *in vivo*, it has been shown that remyelination in this system is dependent on the presence of microglia/macrophages which elicit myelin clearance in short time-frames [53]. In the spheroid cultures, the g-ratio values were comparable with those of other *in vivo *and *in vitro *models [54-57], and were reduced as expect following demyelination. However, the characteristic higher g-ratio values following remyelination seen *in vivo *were not observed in this study, with g-ratios returning to those of pre-demyelinated axons. This indicates that compaction during the remyelination phase differs between the *in vivo *and *in vitro *situation. This could be attributed to a smaller number of lamellar wraps, often coincident with shorted internodal distances. It is also known that axons increase diameter in response to myelination by hyperphosphorylation of neurofilaments. These processes may be altered in a system that has developed and is maintained with no spatial restriction or competition, such as the spheroid model employed here.

It is interesting that, while it was able to modulate the remyelination phase, fingolimod did not have any effect on the initial demyelinative event. This is perhaps not surprising when considering the method of insult. The primary effect of lysophosphotidyl choline on myelin is a toxic event occurring through lipid peroxidation, and this is likely to occur rapidly *in vitro *[58]. The effect on microglia is likely to be slower [59], and therefore microglial activation would peak at a later time-point, following the majority of myelin damage. However, microglial activation persists following this acute insult. We postulate that it is this prolonged microglial activation against which fingolimod is acting to produce enhanced remyelination.

Spheroids are allowed to recover for 11 days in order to monitor remyelination. This relatively short time-period is in excess of other similar published methods: remyelination is seen after 3 [40] or 11 days [60] in slice cultures following transient insult, indicating that these timeframes are applicable. Indeed in animal models using lysophosphotidyl choline, remyelination can be observed in a similar timeframe [61,62]

The aggregating spheroid telencephalon system has proved a useful model of inflammatory demyelination in this study. As well as providing robust myelination and remyelination, the system allows probing of the effects directly on cells of the CNS in the absence of peripheral immune components. In addition, it allows for multi-time point sampling of the same population of cells, and provides the ability for imaging and biochemical/molecular analysis on this same population. However, this study has also detailed some less favorable aspects of the model, namely the lack of correlation of g-ratio values with *in vivo *remyelination. The data presented give some insight into the mechanism of action of demyelination in this paradigm. In the absence of monocytes, microglia and astrocytes are able to produce pro-inflammatory cytokines and reactive oxygen species responsible for demyelination.

The actions of fingolimod on microglial cells indicated here may be extrapolated into the clinical setting, and could result in increased remyelination. In addition to reducing autoimmunity and indirectly allowing natural reparative mechanisms to take place via effects on T-cells, these results indicate that fingolimod may also directly facilitate the remyelinative process through effects on microglia, and thus influence the progression of MS.

## Abbreviations

MS: Multiple sclerosis; CNS: Central nervous system; EAE: experimental autoimmune encephalomyelitis; S1P: Sphingosine 1 phosphate; ERK: Extracellular signal-regulated kinase; OPC: Oligodendrocyte precursor cell; NO: Nitric Oxide; PBS-T: phosphate buffered saline plus 0.1% Tween; MBP: Myelin basic protein; NF: Neurofilament; Fe: Ferritin; TNF-α: Tumor necrosis factor alpha; IL: Interleukin.

## Competing interests

SJ: The author declares that they have no competing interests

DB: The author declares that they have no competing interests

GG: Professor Giovannoni reports having received consulting fees and grant support from Novartis

Fingolimod, BAF312 and AUY954 were provided by Novartis

## Authors' contributions

SJ planned and carried out all experiments, collected and analysed all data, interpreted the data, drafted and edited the manuscript and prepared the figures. GG and DB participated in the study design, coordinated the research, participated in data interpretation and edited the manuscript. All authors read and approved the final manuscript.
